# Modeling the influence of attitudes, trust, and beliefs on endoscopists’ acceptance of artificial intelligence applications in medical practice

**DOI:** 10.3389/fpubh.2023.1301563

**Published:** 2023-11-28

**Authors:** Peter J. Schulz, May O. Lwin, Kalya M. Kee, Wilson W. B. Goh, Thomas Y. T Lam, Joseph J. Y. Sung

**Affiliations:** ^1^Lee Kong Chian School of Medicine, Nanyang Technological University, Singapore, Singapore; ^2^Wee Kim Wee School of Communication and Information, Nanyang Technological University, Singapore, Singapore; ^3^School of Biological Sciences, Nanyang Technological University, Singapore, Singapore; ^4^Center for Biomedical Informatics, Nanyang Technological University, Singapore, Singapore; ^5^Faculty of Medicine, Institute of Digestive Diseases, The Chinese University of Hong Kong, Hong Kong, China

**Keywords:** artificial intelligence, gastroenterology, acceptance, attitudes, trust

## Abstract

**Introduction:**

The potential for deployment of Artificial Intelligence (AI) technologies in various fields of medicine is vast, yet acceptance of AI amongst clinicians has been patchy. This research therefore examines the role of antecedents, namely trust, attitude, and beliefs in driving AI acceptance in clinical practice.

**Methods:**

We utilized online surveys to gather data from clinicians in the field of gastroenterology.

**Results:**

A total of 164 participants responded to the survey. Participants had a mean age of 44.49 (SD = 9.65). Most participants were male (*n* = 116, 70.30%) and specialized in gastroenterology (*n* = 153, 92.73%). Based on the results collected, we proposed and tested a model of AI acceptance in medical practice. Our findings showed that while the proposed drivers had a positive impact on AI tools’ acceptance, not all effects were direct. Trust and belief were found to fully mediate the effects of attitude on AI acceptance by clinicians.

**Discussion:**

The role of trust and beliefs as primary mediators of the acceptance of AI in medical practice suggest that these should be areas of focus in AI education, engagement and training. This has implications for how AI systems can gain greater clinician acceptance to engender greater trust and adoption amongst public health systems and professional networks which in turn would impact how populations interface with AI. Implications for policy and practice, as well as future research in this nascent field, are discussed.

## 1. Introduction

Artificial intelligence (AI) in healthcare has been rapidly developing, with AI applications expanding in clinical settings in almost all medical specialties. Artificial intelligence (AI) has made ground-breaking technological advancements in medical image interpretation (1), diagnosis, risk assessment for conditions (2), and even being used in surgical interventions (3). The trust and acceptance of clinicians is a key factor to integrate AI technology into the healthcare systems (4). However, acceptance of AI is not uniform amongst clinicians. Despite the developments in medical and healthcare-related applications of AI, research studies about the dynamics of drivers of AI acceptance amongst clinicians is limited. This is a significant research gap as clinicians are major stakeholders in the deploy ability of AI. Without the confidence of clinicians, and their acceptance of the use of AI, developments in AI in healthcare and in health systems would be devoid of usability. Given the increasing potential for the utilization of AI in various fields of medicine, further questions arise as to which factors are salient determinants of real-time behaviors pertaining to AI and how these factors contribute to the acceptance of AI amongst clinicians. This research therefore sets out to examine the proposed key drivers (trust, attitude, and beliefs) of AI acceptance in clinical practice, specifically amongst clinicians in the field of gastroenterology, a medical field that is utilizing AI across a broad spectrum of applications, and how the key variables relate to one another.

### 1.1. Conceptual framework

While research on the motivators behind the use and acceptance of AI is limited, research on the drivers of earlier forms of technology acceptance have been scientifically approached in several ways in related disciplines such as communication, information studies and business. These include the utilization of theories such as the theory of reasoned action (TRA) and planned behavior (TPB) which are well-used frameworks for designing interventions (5). TPB posits that the immediate predictor of human behavior is the intention to perform the behavior. This intention is subsequently predicted by key perceptual determinants, namely attitude, subjective norm, and perceived behavioral control towards performing a behavior (6).

Technology Acceptance Models (TAM) (7) explains how people adopt certain technologies. In the public health and medicine space, technology acceptance via TAM models have been used to understand the perspective of service personnel (e.g., doctors), telemedicine (8) and health information websites (9). Central to TAM is the role of trust, which refers to a psychological mechanism driving clinician’s adoption behaviors in clinical environments [e.g., (10)] in medical contexts. Trust is a relevant motivator because medical decisions are characterized by risks and thus require decisions made with confidence. Trust has been proposed as a psychological mechanism driving clinician’s adoption behaviors in medical environments (10). For the purposes of this study, based on these theoretical perspectives, we propose a framework incorporating general attitudes, trust regarding technologies, and beliefs regarding artificial intelligence as the key antecedents for the prediction of AI acceptance intentions. The model is expected to forecast acceptance of AI in terms of willingness to undertake diagnostic and intervention actions in medical practice. The proposed model emphasizes cognitive processes that drive AI acceptance which will motivate AI behaviors such as adoption and usage.

### 1.2. Clinician’s acceptance of AI

While past studies have explored AI perceptions amongst clinicians, there are still numerous gaps in understanding how AI acceptance becomes internalized. Extant studies often explored general or overall levels of trust of their participants. For instance, some questions about trust and acceptance of clinicians include general perceptions such as the problems one sees regarding the application of AI in medicine (11) and whether AI would be a burden for practitioners (12). Other studies report levels of acceptance in a general manner, such as attitudes being “relatively favorable” or an “average” level of acceptance (13). However, little is known about the extent to which overall assessment of AI is driven by trust, beliefs and attitudes towards AI and how these translate into the acceptance of specific AI tools in clinical contexts.

Clinicians’ expectations of AI in performance (i.e., to enhance their job performance) and ease of use may or may not be associated with their intentions to use AI (14). The implementation of a single AI-assisted tool could fail not due to the general attitude but rather due to mistrust in a specific application in the clinical context. One qualitative study found that clinicians expect AI to perform roles in data collection, supporting clinical decisions, advising clinicians, and, in some cases, perform as a fully autonomous system (15). While clinicians may have an expectation of the roles that AI should fulfill, they may also feel that the implementation of AI might have a threat to their identities as clinicians. Another qualitative study found that participants believed that AI implementation could lead to deskilling, removing clinicians from healthcare systems and dehumanize healthcare (16). However, it was also reported in the same study that, on a positive note, some clinicians felt that the implementation of AI could lead to direct and indirect upskilling, leading to better quality of healthcare and more skilled clinicians (16). A general positive attitude may not directly lead to the acceptance of AI. Other factors such as beliefs about AI appear to also influence whether clinicians are willing to accept AI in clinical practice, however these have not been studied along with trust. The impact of trust in specific acceptance of AI, and how beliefs and attitudes to influence user acceptance around AI technology are thus critical gaps that need urgent investigation.

We articulate the hypotheses and research question as follows:

*H1*: Trust, attitudes, beliefs and expectations regarding advantages and disadvantages of AI would influence AI acceptance such that:

- Greater trust will lead to AI acceptance;
- More positive attitudes will lead to greater AI acceptance;
- More positive beliefs regarding AI will increase AI acceptance;
- High positive expectations regarding AI will increase positive attitudes towards AI.

While we expect the three variables to act as antecedents to AI acceptance, the relations between these variables and how they influence AI acceptance are unclear. Following the TRA and TAM frameworks, we expect positive expectancies (including usefulness of AI tools) will impact attitudes towards AI. What is less clear is the path between general attitudes towards AI and the acceptance of single AI applications. Based on the two previously cited theoretical frameworks, attitude would directly predict acceptance of AI. However, as previously mentioned, some studies have shown that a general positive attitude does not always translate into the acceptance of a special AI application. Also, the other relationships between trust, belief and acceptance need clarification. We therefore pose the research question as follows:

*RQ1*: What are the interrelations between trust, attitudes, and beliefs in driving AI acceptance?

## 2. Materials and methods

### 2.1. Research design

Our cross-sectional study utilized a survey of clinicians in the field of gastroenterology. We surveyed clinicians working in the field of gastroenterology. Within the space of gastroenterology, various applications of AI include the identification of lesions (e.g., benign or malignant), detection of lesions, development of objective scoring systems for risk stratification, predicting treatment response, evaluating metrics, and partaking in surgical intervention (17). AI can be implemented to complement existing practices, such as in interpretation of medical imagery in detecting colorectal lesions (17–19). While AI can assist clinicians in their regular practices, in invasive surgical procedures, AI can also be used in place of a clinician, such as in an endoscopy to guide the need of colonoscopic polypectomy (17–19).

We adopted an email survey as the targeted respondents were familiar with online activities and are digitally literate. Clinicians were eligible if they were (i) practicing clinicians in the field of gastroenterology and (ii) practicing medicine in the Asia-Pacific region. Ethical approval was obtained from the university institutional review board.

### 2.2. Procedures

To detect any ambiguous questions, we first pretested the survey with two gastroenterologists. Feedback was gathered from subjects and improvements to the final survey were made using input. For the main survey, each prospective sample unit was identified via regional gastro networks and received an email describing the study and requested their participation prior to the survey’s distribution. From November 2022 to January 2023, eligible participants were contacted via email or WeChat (if they were in China). Upon agreeing to participate in the study, each participant was sent a Qualtrics link to the survey. Participants were instructed and allowed to complete the questionnaire at their own convenience. The questionnaire was administered in the English language. The survey questionnaire included questions about participants’ socio-demographic characteristics, work-related details, perceptions of AI, and acceptance of AI.

### 2.3. Measures

The questionnaire was based on the expectancy-value framework, major constructs of the TPB research framework (6), and the TAM measures (20). We also included items from various validated questionnaires in extant literature for our final questionnaire.

General expectations regarding AI in gastroenterology (*expectp*) in diagnosis and for clinical tasks were measured with seven items adapted from two questionnaires (21, 22). Items included amongst others “The quality of care will increase,” “There will be fewer medical errors,” “AI will improve diagnostic efficiency over the next years,” “AI will enhance patients’ welfare (e.g., patients’ state of doing well in areas such as happiness, well-being, or prosperity),” AI will improve diagnostic efficiency over the next years,” “Computer-assisted polyp detection will improve endoscopic performance,” and were presented on a scale ranging from 1 (Impossible to happen to) to 7 (Certain to happen). These items reflect participants’ expectations of AI, including clinicians perceived usefulness of AI, and perceptions of barriers and facilitators for use of AI, were adapted from three questionnaires in previous literature (22–24). A composite measure presenting an average of the responses was built. Uni-dimensionality was assured via principal component analysis (α = 0.87, M = 5.52, SD = 0.80).

General attitudes towards the use of AI in gastroenterology (*attiai*) was measured based on a semantic differential scale. One question consisted of six items, and asked about participants positive or negative feelings towards AI technology will be used in patient care. Participants rated their feelings between 1 (negative) and 7 (positive). Some examples of the items included “bad/good, worthless/valuable, harmful/beneficial” (α = 0.90, M = 5.80, SD = 0.84).

Trust in AI and the results it can produce (*trust*) was based on four items. The items were adapted from an existing questionnaire (25). Some examples of the items included “If tested properly, the findings of AI can be trusted” an “If licensed properly, the findings of AI can be trusted.” 7-point Likert scales were presented, and the measure used was an average of the responses. Uni-dimensionality was again assured via principal component analysis (α = 0.83, M = 5.49, SD = 0.82).

The two remaining variables in our model, namely belief and acceptance, pertain to the presentation of three real AI scenarios which we developed to test the two concepts within a specific AI application in gastroenterology. We developed these three AI scenarios related to the application of AI in current practice in gastroenterology. In particular, we focused on colonoscopy and management of colorectal polyps, which are already proven as being, on average, more reliable than clinicians’ judgment (18, 19). The scenarios were presented, in order of ascending difficulty. To illustrate the nature of the scenarios, the first one is provided here as an example: “Imagine you are attending an informal meeting of colleagues. Your colleagues are not experts in AI and have about the same amount of understanding as you do. The conversation turns to innovation in medicine, especially machine learning algorithms and their potential to assist in the interpretation of medical imagery in the early detection of colon cancer. One of the colleagues speaks about a patient who underwent a colonoscopy assisted by a machine learning algorithm. When the algorithm indicated that the patient had a colonic polyp, the colleague asked for an additional biopsy. It turned out that the result produced by the algorithm was correct.”

After each scenario, participants rated the extent to which they would believe and accept the use of AI in these three procedures with increasing degrees of risk. Scenario 2 described an instance where AI could be used to classify and characterize colonic polyps as either neoplastic (adenomatous polyp) which requires treatment or non-neoplastic (hyperplastic polyp) which does not mandate removal. Scenario 3 described an instance where AI could be used to removal of polyp by using an endoscopic device (snare) in endoscopic polypectomy. Belief was measured in all three scenarios asking participants whether they fully believe or harbor doubts regarding the colleague who reported in each of the scenarios about the successful application of a machine learning algorithm. These three items presented in a 7-point scale were composed in a single measure and averaged (α = 0.75, M = 5.39, SD = 0.92).

The dependent variable acceptance of AI (accept) is based on two items asked in all three scenarios (six items in total). These questions asked on a 7-point scale ranging from 1 (“Having major doubts”) to 7 (Fully believe), “Do you believe that machine learning algorithm can, in some cases (as the one described), perform better than human beings?” and “Will you accept the method?” Uni-dimensionality was verified using principal components analysis and reliability was assessed by Cronbach alpha (α = 0.89, M = 5.45, SD = 0.90).

### 2.4. Data analysis

Data analysis was conducted using Mplus Version 8.10. Participants’ socio-demographic characteristics, job-specific details, current use of AI, and intention to use AI were summarized using descriptive statistics such as median and inter-quartile range (IQR).

## 3. Results

### 3.1. Participant socio-demographics

A total of 164 participants responded to the survey. Participants had a mean age of 44.49 (SD = 9.65). Most participants were male (*n* = 116, 70.30%) and specialized in gastroenterology (*n* = 153, 92.73%). Participants had a variety of clinical experiences, but slightly more than half worked more than 10 years (*n* = 93, 56.36%) and worked with more than 20 other staff members (*n* = 83, 50.30%). Most were consultants or senior consultants (*n* = 111, 67.280%), and worked in public hospitals (*n* = 110, 66.67%). The participant demographics are summarized in Table 1.

**Table 1:** Participant demographics and general characteristics.

	Total (*N* = 164)
	**Mean (SD)**
**Age (years)**	43.96 (9.45)
	***n*** **(%)**
**Gender**	
Male	107 (65.24)
Female	37 (22.56)
Undisclosed	20 (12.20)
**Years practicing in specialty**	
Less than 5 years	38 (23.17)
5–10 years	30 (18.29)
11–20 years	46 (28.05)
Over 20 years	38 (23.17)
Undisclosed	12 (7.32)
**Department size**	
Less than 5 staff members	9 (4.49)
5–10 staff members	28 (17.07)
11–20 staff members	36 (21.95)
Over 20 staff members	79 (48.17)
Undisclosed	12 (7.32)
**Have used AI in Work**	
Yes	111 (67.68)
No	51 (31.10)
Undisclosed	2 (1.22)
**Know other clinicians who use AI**	
Yes	132 (80.49)
No	32 (19.51)
Undisclosed	0 (0)

### 3.2. Relationships between expectations, trust, and acceptance of AI

Table 2 provides detailed information about the continuous variables used in the model, including means and standard deviations, skewness, and kurtosis. The median values for each variable – not reported in the Table 2 – closely resemble the mean values. Following the parameters outlined by Kline (26) and Finney and DiStefano (27, 28), all absolute univariate skewness and kurtosis values remain within acceptable thresholds. Specifically, no skewness values surpass the absolute value of 2.0, and none of the kurtosis values exceed the absolute value of 7.0.

**Table 2:** Descriptive statistics, alpha coefficients, composite reliabilities, and correlations.

Variable	M	SD	Skewness	Kurtosis	No of items	a	1	2	3	4
Expectations	5.52	0.80	−0.36	0.35	7	0.871				
Attitude	5.80	0.84	−0.73	0.19	6	0.901	0.47**			
Trust	5.49	0.82	−0.73	1.31	4	0.832	0.62**	0.54**		
Belief	5.39	0.92	−0.33	−0.32	3	0.747	0.42**	0.49**	0.42**	
Acceptance	5.45	0.90	−0.30	0.27	6	0.887	0.48**	0.49**	0.48**	0.80**

*N* = 164 Clinicians in the field of gastroenterology. ** Correlation significant at *p* < 0.01 (two-tailed test).

### 3.3. Outliers

A leverage score was calculated for each participant to identify possible non-model-based outliers, defined as individuals with a leverage score three times higher than the mean (29). No outliers were identified in this analysis. To identify model-based outliers, we examined the DfBeta values for each respondent, considering a DfBeta value exceeding three times the standard error of the coefficient as indicative of an outlier. Based on this analysis, no outliers were detected.

### 3.4. Missing data and data imputation

Initially, a missing value cutoff was applied, whereby any sample containing more than 40% of missing values in all questions was excluded from the dataset. Subsequently, a hard cutoff filter was implemented, whereby any sample missing data for any of the outcome variables was excluded from the dataset. This rigorous approach to data filtering aims to minimize bias and increase the robustness of subsequent analyses, ensuring accurate and reliable results. To impute missing values in this dataset, we used KNNImputer. This is a machine learning-based imputation method that estimates missing values by using a nearest-neighbor approach. It calculates the distance between samples based on their feature values and imputes missing values based on the values of their nearest neighbors. In this study, the default *k* = 5 was used, meaning that KNNImputer used the 5 closest observations to estimate each missing value.

### 3.5. Non-normality

To test whether the basic assumption of multivariately normally distribution of variables was met (as it is required for maximum likelihood analysis), we used the Mardia test for multivariate skewness and kurtosis.^1^ A statistically significant result emerged for kurtosis, indicating non-normality at the multivariate level. The troublesome kurtosis level is evident for the measure of trust. We, therefore, tested the parameter estimation in three different approaches. First, we conducted a maximum likelihood analysis; second, we used the maximum likelihood with robust standard errors (MLR) in MPlus. In the third scenario we applied a bootstrapping approach, conducting 10,000 bootstrap replications, estimating standard errors, *p* values, and confidence intervals. Comparing the results of all three approaches did not lead to any major difference. In the following, we will report results from the maximum likelihood analysis.

For the data analysis, we used the software Mplus Version 8.10. To validate the model, we relied on the traditional absolute fit indices (chi-square, Root Mean Square Error of Approximation [RMSEA], and Standardized Root Mean Square Residuals [SRMR]) and incremental fit indices (Comparative Fit Index [CFI], and Tucker-Lewis Index [TFI]). We calculated the statistical power for the tests of the path coefficients necessary to consider possible Type II errors for statistically non-significant path coefficients using an *a-priori* sample size calculator for SEM (30). It turned out that our sample size is sufficient for detecting possible Type II errors.

### 3.6. Model test

**Figure 1:**
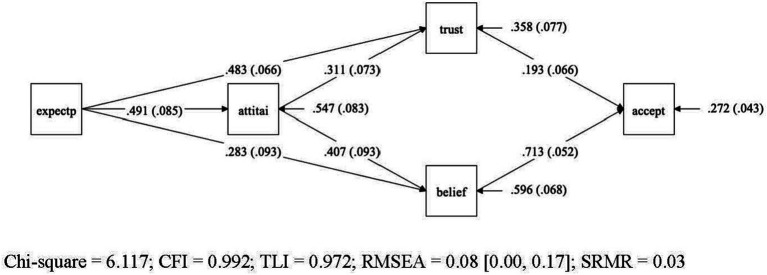
Final model for the acceptance model – graphical representation of final model for acceptance of AI-specific applications in gastroenterology. Variables: General expectations regarding AI (expectp), general attitudes towards AI (attiai), trust in AI (trust), beliefs in AI (belief), acceptance of AI (accept). Legend: Straight lines represent the presumed causal paths, rectangles denote measured variables, numbers in the mid of the line are unstandardized path coefficients, values in brackets represent their standard errors, the values pointing to each endogenous construct indicate the residual variance of each construct with their standard error in brackets.

The initial model we tested with Mplus version 8.10 is similar to the final model presented in Figure 1, except that in the initial model we included a direct path between general attitude towards AI and acceptance of specific AI applications in Gastroenterology. All other variables and paths were included in both the initial and final model.

The initial model yielded a marginal significant chi-square value (*p* = 0.09), indicating a poor model fit. Also, other traditional indices, such as RMSEA = 0.093 and TLI = 0.962 suggested a mixed picture. Inspection of the model diagnostics indicated that the source of ill fit was the assumption of the direct path between general attitude towards AI and acceptance of AI specific applications in gastroenterology. The direct path proved to be non-significant (b = 0.069, *p* = 0.266). Once this path between attitude and acceptance was dropped from the initial model, the model yielded a good fit, and the traditional indices of overall model fit were satisfactory (c2 = 6.117, df = 3, *p* < 0.106, RMSEA = 0.080, CFI = 0.992, TLI = 0.972, SRMR = 0.028). The examination of the single path coefficients also yielded satisfactory results, all statistically significant (*p* < 0.01). The single unstandardized coefficients are reported in the figure of the model (Figure 1), while Table 2 presents the confidence intervals from the bootstrap analysis for the unstandardized coefficients of all four-mediator path as well as the effect size of the model.

As convention dictates, we examined next the covariance and mean residuals as measures of local model fit. In Mplus the standardized residuals for covariances are provided as z-scores and all z-scores were below 1.46. Therefore, this more detailed inspection of model diagnostics also revealed a good model fit. Next, we included modification indices to examine whether the model would further improve if additional restrictions were added, or existing paths were dropped. No indices revealed any greater potential gain by adding or dropping any path of the final model.

Hypotheses 1 (H1) proposed that greater trust (H1a), positive beliefs (H1b), and positive attitude (H1c) are associated with higher level of AI acceptance. This hypothesis was partially supported. While greater trust and positive beliefs are directly linked to AI acceptance, positive attitude did not impact directly on the AI acceptance but does so only via the two mediators trust and belief. This result confirms what previous studies have shown, namely, that a general positive attitude does not allow any conclusions about whether a person will also accept a specific AI application. In the baseline model we tested this direct path, and it turned out to be not significant. On the other side, the result differs from what the Theory of Reasoned Action and the Technology Acceptance Model would predict, namely a direct path from attitude to acceptance.

Table 3 shows the 95% Confidence intervals for unstandardized path coefficients. Regarding the RQ regarding the relations holding between expectations, attitude, trust, belief, and acceptance, positive expectations towards AI do not only predict a positive general attitude towards AI. They are also found to be associated with higher levels of trust and beliefs in AI which, in turn, impacts on the acceptance of AI. All four mediator paths (*cf.* Table 3) taken together sum up to 47% of explained variance in AI acceptance in our model.

**Table 3:** 95% Confidence intervals for unstandardized path coefficients.

	Effect	BootSE	BootLLCI	BootULCI	*p* value
Total	0.467	0.0813	0.3094	0.6308	<0.001
Expectations = > beliefs = > accept	0.202	0.067	0.072	0.341	<0.001
Expectations = > trust = > accept	0.093	0.034	0.032	0.169	<0.001
Expectations = > attitude = > beliefs = > accept	0.142	0.042	0.070	0.236	<0.001
Expectations = > attitude = > trust = > accept	0.030	0.015	0.008	0.065	<0.01

For single paths the coefficient is reported in the model above.

## 4. Discussion

The research aimed to uncover how trust, attitudes towards AI and beliefs regarding AI influence AI acceptance by medical practitioners who are key stakeholders in the health system. This was articulated in Hypotheses Set 1 and Research Question 2. To that end, our study tested a sequential model of AI application acceptance where acceptance of specific AI applications in medical practice is presented as a function of four antecedents: general expectations towards the usefulness of AI, attitudes towards AI, and trust and belief in existing AI applications in gastroenterology. Structural Equation Modeling based methods were used to elaborate on the psychological mechanisms underlying this acceptance and the paths that lead to the acceptance. The results of the analysis are consistent with our hypotheses in several ways but also revealed unique features in the cognitive processes. Contrary to a general assumption, there is no direct path from general attitudes in artificial intelligence to the acceptance of existing clinical AI applications. Instead, the acceptance of individual applications depends on trust in their respective efficiency as well as beliefs regarding AI.

First, based on the TRA framework (6), we predicted that a general positive attitude towards AI is a function of positive expectations regarding its usefulness. According to the same framework, we should have expected that acceptance of AI applications is predicted by a positive attitude. This, however, was not the case. Our findings showed that clinicians accept and are willing to employ specific AI applications if they are convinced that a specific application is trustworthy and useful in a specific clinical context. It is not necessary that a general positive attitude towards AI will also translate into a willingness to apply specific AI tools. Rather, it is likely that familiarity with specific tools gained by acquired experiential knowledge is key to the acceptance of AI tools. To enhance AI utilization by clinicians, one potential translational approach would be to encourage newer and less experienced clinicians to achieve awareness of and make acquaintance with the AI applications. However, our findings show that trust and beliefs need to go hand in hand with exposure to AI. Therefore, introductions to AI need to be more effectively done through trust enhancers, such as involving trusted professional sources, including senior gastroenterologists and surgeons. In terms of the future implementation of proven AI-supported tools in everyday clinical practice, this primarily means that experiential training based on knowledge gained from trusted and highly credible user experience is required, which can lead to a higher acceptance of AI tools.

Similarly, our findings differ also from the other theoretical model, namely the TAM, in an important way. The TAM model suggests that the adoption of an innovative technology is based on consumer attitudes, which in turn are predicted by perceived usefulness and perceived ease of use. Consistent with this model, we found that attitude is predicted by positive expectations such as perceived usefulness and ease of use of AI; however, whether the attitude impacts on the acceptance of AI was mediated by the trust and belief in the efficacy of a specific AI tool. Furthermore, we found in our model that general positive expectations towards AI directly exert impact on beliefs and trust in the specific AI tools which in turn affect its acceptance.

## 5. Implications, limitations, and conclusions

This research aimed to provide knowledge regarding how the antecedents, namely trust, attitude, and beliefs, drive AI acceptance in clinical practice via a conceptual model.

The findings showed that the proposed model was capable of explaining the influencers and processes behind AI acceptance by clinicians in the field of gastroenterology who are key stakeholders in public health systems. The proposed model provides base framework to better understand how professionals in the field can come to accept or reject AI in medical practice. Furthermore, our results show that the relationship between expectations of and acceptance in AI is not direct as hypothesized but is mediated through trust and knowledge of artificial acceptance. The findings have implications for guiding AI educational approaches in teaching AI applications, and for general assimilation of AI tools into health and medical practice.

As AI becomes increasingly used as part of regular healthcare practice, we anticipate that (a) healthcare professions will need to take courses or training and (b) the curriculum for medical students will be updated. To understand and utilized AI that is being implemented in healthcare, healthcare professionals should be required to take specialized training. This way they can incorporate the AI into regular practice and feel confident in making AI-related recommendations to their patients. In terms of implications for medical education, the curriculum for specialties where AI is being incorporated as regular practice will be updated to keep up with new medical practices. We also anticipate that digital literacy will improve as healthcare practitioners partake in more training, and with the updated curriculum for students. With the vast amount of AI software being developed, it will also be important for healthcare professionals to know about the software that will be utilized. New AI and systems are constantly being developed and applied in various fields of healthcare, such as in breast cancer medical imaging (31, 32), tracking patient’s medical history (33), and patient management systems (34). In educating both present and future healthcare practitioners, the acceptance of AI can be cultivated. However, we recognize that, currently, there is no single set of regulations that developers must abide by. However, we recognize that, currently, there is no single set of regulations that developers must abide by. A number of countries have recognized potential ethical issues and AI and have convened high level committees working towards regulations or guidelines. Future studies are recommended to the opinions of health authorities and potentially other stakeholders involved in policy making, towards the ethical considerations in using AI in regular practice.

Beyond the implications of our findings for healthcare and practice, there are consequences for public health. The burden of disease is increasing yearly. For instance, in the USA, nearly two million new cancer cases and over half a million cancer deaths are projected to occur in 2023 (35). Cancer screening, including those that use AI, can be used to detect cancer early, to detect a larger number of cancer cases. With the implementation of AI, delivery of healthcare services may change drastically. Members of the public are likely to look to clinicians and their perspectives as trustworthy sources of information. Thus, understanding clinicians trust and accept AI will be beneficial for all populations. Furthermore, findings from this study can aid in the development of guidelines for the clinician use of AI in healthcare, an important enabler of greater public trust in technology use in healthcare settings. Our findings on the trust and acceptance of AI may not be entirely generalizable to the global population, as participants are from the Asia-Pacific Region. We recommend that future studies with different populations should be conducted to identify factors associated with the trust and acceptance of AI. Another study that utilized a research model with ten constructs to determine intention to use intelligent agents in medical imaging had a similar suggestion (36). They recommended that data collection of more clinical characteristics may be beneficial to validate models (36). They also recommended that constructs, such as trust and intention, should be measured in scenario-specific cases, which we have done for the purposes of our study. While our participant population may not be representative of the global gastroenterologist population, our model reflects how variables are related to each other, and contributes important findings to the field.

Another option to enhance the confidence of the stakeholders and publics would be for the AI resources to be certified or recognized by credible organizations such as the local health authorities. An added approach in medical education and coaching involves going beyond texts or user manuals to carefully guiding doctors through experiential learning or via immersive courses where they can witness AI in action and its outcome real time. Such an educational strategy would allow the slow buildup of trust and to nudge positive beliefs regarding AI. From a broader application perspective, a further set of considerations pertains to the design of AI tools. As AI technology needs to work alongside humans in a trusted and effective manner, the design of AI applications requires the involvement of human-centered approaches. This involves engaging appropriate stakeholders in the developmental journey, especially the clinicians themselves. Human-centered AI approaches which would involve a holistic understanding of health systems (37) should be encouraged early in the design of tools and services. AI is also continually being updated and developed, future studies should be conducted regarding regulations surrounding the development and implementation of AI. In particular, policymakers and researchers in the field should be interviewed or surveyed to better understand their opinions towards the implementation of AI in healthcare.

This study also has some limitations. For one, our survey was conducted only amongst clinicians in the field of gastroenterology. While the findings are likely to be applicable for some other medical fields, clinicians in other specialties may have divergent considerations and display other directions of behaviors in dissimilar medical environments. Hence, future research should examine replication potential in other types of medical specializations (e.g., mental health) and to a wider population of healthcare workers. Second, our survey participants were from Asia-Pacific countries, and hence may be bound by cultural characteristics which differ from other regions in the world. Future research should thus broaden the sample base to more global regions. Third, our study also looked at trust and attitude in a general sense, and in specific scenarios. While we asked participants a wide variety of questions, future studies are recommended to delve deeper into specific variables of interest. For instance, the ethics of applying AI in the clinical context will impact all stakeholders, including developers, clinicians, and patients. It would be beneficial to conduct qualitative and quantitative studies to understand their opinions towards the ethics, and the potential repercussions, of using AI in healthcare. Fourth, our study has an implicit assumption – that the applications of AI can be trusted. The scenarios that we presented to our participants in the questionnaire were based on existing applications of AI that have been, or are being, tested in gastroenterology practice. In this sense, the AI that is presented in our study have been tested and can be trusted to be reliable. However, we recognize that the extent to which a machine (or AI) should be trusted will become more of an issue with the development and integration of AI in healthcare.

In summary, this research sought to model the antecedents of AI acceptance in clinical practice. Our findings showed that attitudes towards AI had little direct impact on acceptance, but indirectly influences behaviors through trust and beliefs. The role of trust and beliefs as primary mediators suggest the these should be areas of focus in AI education, familiarization and training. This has implications for how AI systems can gain greater clinician acceptance to engender greater trust and adoption amongst public health systems and professional networks. AI has the strong potential to improve healthcare both in the region and globally, and hence more research is needed to understand better the acceptance and avoidance of AI deployment in healthcare.

## Data availability statement

The raw data supporting the conclusions of this article will be made available by the authors, without undue reservation.

## Author contributions

PS: Conceptualization, Data curation, Formal analysis, Methodology, Writing – original draft, Writing – review & editing. ML: Conceptualization, Data curation, Formal analysis, Methodology, Writing – original draft, Writing – review & editing. KK: Conceptualization, Formal analysis, Writing – original draft, Writing – review & editing. WG: Data curation, Formal analysis, Writing – review & editing. TL: Investigation, Project administration, Resources, Writing – review & editing. JS: Funding acquisition, Project administration, Supervision, Writing – review & editing.

## Footnotes

^1^ https://webpower.psychstat.org/models/kurtosis/

## References

[1] Elmore JG Lee CI. Artificial intelligence in medical imaging-learning from past mistakes in mammography. JAMA Health Forum. (2022) 3:e215207. doi: 10.1001/jamahealthforum.2021.5207, PMID: 36218833 PMC9648493

[2] Ren Y Loftus TJ Datta S Ruppert MM Guan Z Miao S. Performance of a machine learning algorithm using electronic health record data to predict postoperative complications and report on a mobile platform. JAMA Netw Open. (2022) 5:e2211973. doi: 10.1001/jamanetworkopen.2022.11973, PMID: 35576007 PMC9112066

[3] Loftus TJ Tighe PJ Filiberto AC Efron PA Brakenridge SC Mohr AM. Artificial intelligence and surgical decision-making. JAMA Surg. (2020) 155:148–58. doi: 10.1001/jamasurg.2019.4917, PMID: 31825465 PMC7286802

[4] Asan O Bayrak AE Choudhury A. Artificial intelligence and human trust in healthcare: focus on clinicians. J Med Internet Res. (2020) 22:e15154. doi: 10.2196/15154, PMID: 32558657 PMC7334754

[5] Taylor D Bury M Campling N Carter S Garfied S Newbould J. A review of the use of the health belief model (HBM), the theory of reasoned action (TRA), the theory of planned behaviour (TPB) and the trans-theoretical model (TTM) to study and predict health related behaviour change. London, UK: National Institute for Health and Clinical Excellence (2006).

[6] Ajzen I. The theory of planned behavior. Organ Behav Hum Decis Process. (1991) 50:179–211. doi: 10.1016/0749-5978(91)90020-T

[7] Davis FD. Perceived usefulness, perceived ease of use, and user acceptance of information technology. MIS Q. (1989) 13:319–40. doi: 10.2307/249008

[8] Chau PYK Hu PJ. Examining a model of information technology acceptance by individual professionals: an exploratory study. J Manag Inf Syst. (2002) 18:191–229. doi: 10.1080/07421222.2002.11045699

[9] Kim D Chang H. Key functional characteristics in designing and operating health information websites for user satisfaction: an application of the extended technology acceptance model. Int J Med Inform. (2007) 76:790–800. doi: 10.1016/j.ijmedinf.2006.09.001, PMID: 17049917

[10] Asan O Yu Z Crotty BH. How clinician-patient communication affects trust in health information sources: temporal trends from a national cross-sectional survey. PLoS One. (2021) 16:e0247583. doi: 10.1371/journal.pone.0247583, PMID: 33630952 PMC7906335

[11] AlZaabi A AlMaskari S AalAbdulsalam A. Are physicians and medical students ready for artificial intelligence applications in healthcare? Digit Health. (2023) 9:20552076231152167. doi: 10.1177/20552076231152167 36762024 PMC9903019

[12] Swed S Alibrahim H Elkalagi NKH Nasif MN Rais MA Nashwan AJ. Knowledge, attitude, and practice of artificial intelligence among doctors and medical students in Syria: a cross-sectional online survey. Front Artif Intell. (2022) 5:1011524. doi: 10.3389/frai.2022.1011524, PMID: 36248622 PMC9558737

[13] Hamedani Z Moradi M Kalroozi F Manafi Anari A Jalalifar E Ansari A. Evaluation of acceptance, attitude, and knowledge towards artificial intelligence and its application from the point of view of physicians and nurses: a provincial survey study in Iran: a cross-sectional descriptive-analytical study. Health Sci Rep. (2023) 6:e1543. doi: 10.1002/hsr2.1543, PMID: 37674620 PMC10477406

[14] Tran AQ Nguyen LH Nguyen HSA Nguyen CT Vu LG Zhang M. Determinants of intention to use artificial intelligence-based diagnosis support system among prospective physicians. Front Public Health. (2021) 9:9. doi: 10.3389/fpubh.2021.755644 PMC8661093 34900904

[15] Amann J Vayena E Ormond KE Frey D Madai VI Blasimme A. Expectations and attitudes towards medical artificial intelligence: a qualitative study in the field of stroke. PLoS One. (2023) 18:e0279088. doi: 10.1371/journal.pone.0279088, PMID: 36630325 PMC9833517

[16] Aquino YSJ Rogers WA Braunack-Mayer A Frazer H Win KT Houssami N. Utopia versus dystopia: professional perspectives on the impact of healthcare artificial intelligence on clinical roles and skills. Int J Med Inform. (2023) 169:104903. doi: 10.1016/j.ijmedinf.2022.104903, PMID: 36343512

[17] Kröner PT Engels MML Glicksberg BS Johnson KW Mzaik O van Hooft JE. Artificial intelligence in gastroenterology: a state-of-the-art review. World J Gastroenterol. (2021) 27:6794–824. doi: 10.3748/wjg.v27.i40.6794, PMID: 34790008 PMC8567482

[18] Xu P Zhu X Clifton DA. Multimodal learning with transformers: a survey. IEEE Trans Pattern Anal Mach Intell. (2023) 45:12113–32. doi: 10.1109/TPAMI.2023.3275156 37167049

[19] Sung JJ Poon NC. Artificial intelligence in gastroenterology: where are we heading? Front Med. (2020) 14:511–7. doi: 10.1007/s11684-020-0742-4, PMID: 32458189

[20] Davis FD. A technology acceptance model for empirically testing new end-user information systems: theory and results (Doctoral dissertation). Massachusetts Institute of Technology Sloan School of Management, Cambridge, MA. (1985).

[21] Hah H Goldin DS. How clinicians perceive artificial intelligence-assisted technologies in diagnostic decision making: mixed methods approach. J Med Internet Res. (2021) 23:e33540. doi: 10.2196/33540, PMID: 34924356 PMC8726017

[22] Wadhwa V Alagappan M Gonzalez A Gupta K Brown JRG Cohen J. Physician sentiment toward artificial intelligence (AI) in colonoscopic practice: a survey of US gastroenterologists. Endosc Int Open. (2020) 8:E1379–84. doi: 10.1055/a-1223-1926, PMID: 33015341 PMC7508643

[23] Sarwar S Dent A Faust K Richer M Djuric U van Ommeren R. Physician perspectives on integration of artificial intelligence into diagnostic pathology. NPJ Digit Med. (2019) 2:28. doi: 10.1038/s41746-019-0106-0, PMID: 31304375 PMC6550202

[24] Walter Z Lopez MS. Physician acceptance of information technologies: role of perceived threat to professional autonomy. Decis Support Syst. (2008) 46:206–15. doi: 10.1016/j.dss.2008.06.004

[25] Jungmann F Jorg T Hahn F Pinto dos Santos D Jungmann SM Düber C. Attitudes toward artificial intelligence among radiologists, IT specialists, and industry. Acad Radiol. (2021) 28:834–40. doi: 10.1016/j.acra.2020.04.011, PMID: 32414637

[26] Kline RB. Principles and practice of structural equation modeling. 3rd 3rd ed. New York, NY, US: Guilford Press (2011).

[27] Finney S.J. DiStefano C., (2013). Nonnormal and categorical data in structural equation modeling. Structural equation modeling: a second course, 2nd ed. Charlotte, NC, US: IAP Information Age Publishing. 439–492.

[28] Finney SJ DiStefano C. Non-normal and categorical data in structural equation modeling. Struct Equ Model. (2006) 10:269–314.

[29] Jaccard J Wan C. LISREL approaches to interaction effects in multiple regression. California: Thousand Oaks (1996).

[30] Soper D. (2023). A-priori sample size calculator for structural equation models. Available at: https://www.danielsoper.com/statcalc/calculator.aspx?id=89.

[31] Calisto FM Santiago C Nunes N Nascimento JC. BreastScreening-AI: evaluating medical intelligent agents for human-AI interactions. Artif Intell Med. (2022) 127:102285. doi: 10.1016/j.artmed.2022.102285, PMID: 35430044

[32] Morais M. Calisto FM Santiago C Aleluia C Nascimento JC. (2023). Classification of breast cancer in Mri with multimodal fusion. 2023 IEEE 20th international symposium on biomedical imaging (ISBI). 1–4.

[33] Nashwan AJ Abujaber AA Choudry H. Embracing the future of physician-patient communication: GPT-4 in gastroenterology. Gastroenterol Endosc. (2023) 1:132–5. doi: 10.1016/j.gande.2023.07.004

[34] Akhlaghi H Freeman S Vari C McKenna B Braitberg G Karro J. Machine learning in clinical practice: evaluation of an artificial intelligence tool after implementation. Emerg Med Australas. (2023). doi: 10.1111/1742-6723.14325 37771067

[35] Siegel RL Miller KD Wagle NS Jemal A. Cancer statistics, 2023. CA Cancer J Clin. (2023) 73:17–48. doi: 10.3322/caac.21763, PMID: 36633525

[36] Calisto FM Nunes N Nascimento JC. Modeling adoption of intelligent agents in medical imaging. Int J Hum Comput Stud. (2022) 168:102922. doi: 10.1016/j.ijhcs.2022.102922

[37] Bajwa J Munir U Nori A Williams B. Artificial intelligence in healthcare: transforming the practice of medicine. Future Healthc J. (2021) 8:e188–94. doi: 10.7861/fhj.2021-0095, PMID: 34286183 PMC8285156

